# Preoperative Collateral Perfusion Using Arterial Spin Labeling: A Predictor of Surgical Collaterals in Moyamoya Angiopathy

**DOI:** 10.3389/fnins.2022.839485

**Published:** 2022-03-14

**Authors:** Maoxue Wang, Yi Wang, Wen Zhang, Xiance Zhao, Yongbo Yang, Bing Zhang

**Affiliations:** ^1^Department of Radiology, The Affiliated Drum Tower Hospital of Nanjing University Medical School, Nanjing, China; ^2^Department of Neurosurgery, The Affiliated Drum Tower Hospital of Nanjing University Medical School, Nanjing, China; ^3^Philips Healthcare, Shanghai, China; ^4^Institute of Brain Science, Nanjing University, Nanjing, China

**Keywords:** arterial spin labeling, collateral circulation, revascularization, moyamoya angiopathy, predictors

## Abstract

**Objectives:**

Various degrees of surgical collateral circulation are often found in moyamoya angiopathy (MMA) patients after revascularization. Little is known about arterial spin labeling (ASL) that affects surgical collateral circulation. This study aimed to investigate the effect of ASL on surgical collaterals in patients with MMA after combined bypass surgery.

**Methods:**

MMA patients with complete radiological and clinical information, who had undergone combined bypass, were enrolled in this study. Surgical collaterals were classified as good or poor based on the Matsushima standard. Cerebral perfusion on ASL was quantitatively analyzed as relative cerebral blood flow (rCBF). The qualitative collateral score was calculated using a four-grade scale. Univariable and multivariable logistic regressions were performed to identify the predictors for surgical collaterals after combined bypass.

**Results:**

In total, 66 hemispheres of 61 patients (47 years old ± 8.66) were prospectively included (29 and 37 hemispheres with good and poor surgical collaterals, respectively). The presurgical collateral score was significantly lower in patients with good surgical collaterals (13.72 scores ± 7.83) than in those with poor surgical collaterals (19.16 scores ± 6.65, *P* = 0.005). The presurgical rCBF and modified Rankin scale (mRS) scores were not significantly different between the two groups (P_rCBF_ = 0.639, P_mRS_ = 0.590). The collateral score was significantly elevated (good: 13.72 scores ± 7.83 *vs*. 20.79 scores ± 6.65, *P* < 0.001; poor: 19.16 scores ± 6.65 *vs*. 22.84 scores ± 5.06, *P* < 0.001), and the mRS was reduced (good: 1.66 scores ± 1.14 *vs*. 0.52 scores ± 0.83, *P* < 0.001; poor: 1.49 scores ± 0.90 *vs*. 0.62 scores ± 0.76, *P* < 0.001) in patients after revascularization. Multivariable logistic regression showed that preoperative collateral scores [odds ratio (OR): 0.791; 95% confidence interval (CI): 0.695, 0.900; *P* < 0.001], age (OR: 0.181; 95% CI: 0.039, 0.854; *P* = 0.031), sex (OR: 0.154; 95% CI: 0.035, 0.676; *P* = 0.013), and hypertension (OR: 0.167; 95% CI: 0.038, 0.736; *P* = 0.018) were predictors of surgical collaterals after combined revascularization.

**Conclusion:**

The preoperative collateral score based on ASL could be a predictor for surgical collaterals in patients with MMA after combined bypass surgery. Combined with age, sex, and hypertension, it may have a better predictive effect.

## Introduction

Moyamoya angiopathy (MMA) is a chronic and progressive cerebrovascular disease that is characterized by stenosis of the distal internal carotid artery (ICA), proximal, middle cerebral artery, and anterior cerebral artery. Ischemic stroke, cerebral hemorrhage, headache, and dizziness often occur in patients with MMA (Herve et al., 2018). Revascularization, including direct bypass, indirect bypass, and combined bypass, was recommended to reduce the risk of cerebrovascular events by improving cerebral perfusion (Miyamoto et al., 2014; Zhang et al., 2020). Direct and combined bypass was often used in adults; in addition, the combined bypass had the advantages of both direct and indirect bypass (Kronenburg et al., 2014; Esposito et al., 2018).

Various degrees of surgical collateral circulation are often found in MMA patients after revascularization. Good surgical collateral circulation has been found to be associated with better long-term outcomes compared with the poor group (Zhao et al., 2019). Hemorrhagic onset and ICA moyamoya vessels have an important role in the formation of surgical collateral circulation in MMA patients after indirect bypass surgery (Zhao et al., 2019). Moreover, presurgical collateral stage, p.R4810K variant, and age were also found to be correlated with good surgical collateral circulation in MMA patients after 10 years of indirect bypass surgery (Wang Q. N. et al., 2021).

The presurgical collateral stage was evaluated in a previous study according to the Suzuki stage and leptomeningeal system, which need to be based on digital subtraction angiography (DSA; Liu et al., 2019; Wang Q. N. et al., 2021). However, DSA is an invasive and radiative exam that requires contrast media. Arterial spin labeling (ASL) is a noninvasive magnetic resonance (MR) imaging method without radiation and contrast media, which uses hydrogen protons in the blood as endogenous tracers (Hernandez-Garcia et al., 2019). Three-dimensional (3D) pseudo-continuous ASL (pCASL) was recommended while considering the sufficient image quality (Alsop et al., 2015).

Recently, 4D magnetic resonance angiography (MRA) based on ASL had been shown to have a high consistency with DSA in the presence of distal collaterals in MMA patients (Togao et al., 2018; Wang M. et al., 2021). In addition, ASL can also evaluate cerebral perfusion changes and predict the intensity of collateral flow in patients with MMA compared with DSA (Zaharchuk et al., 2011; Lee et al., 2018). However, little is known about the factors based on ASL that affect surgical collateral circulation after combined bypass surgery.

The purpose of this study was to investigate the effect of ASL on surgical collateral circulation in patients with MMA after combined bypass surgery.

## Materials and Methods

The local institutional review board approved the study protocol before trial initiation (2021-026-02). Written consent was obtained from all subjects before the examinations.

### Subjects

Moyamoya angiopathy patients in a single center from December 2019 to December 2021 were enrolled in this prospective study. Inclusion criteria included the following: (1): MMA patients were diagnosed on MRA or DSA; (2) pre- and postoperative ASL, postoperative super-selective 4D MRA, and susceptibility-weighted imaging (SWI) was acquired; (3) the follow-up time was more than 3 months; and (4) all patients underwent combined bypass surgery. The exclusion criteria were as follows: (1) postoperative intracranial hemorrhage occurring at the anastomosis; (2) motion artifacts on MR images; and (3) contraindications for MR exams. Hypertension, hyperlipidemia, diabetes, history of smoking, drinking, onset type, and pre- and postoperative mRS were collected. The time interval of the preoperative MR exam to bypass surgery and the follow-up time were acquired.

### Magnetic Resonance Imaging

Magnetic resonance images were acquired on a 3.0T scanner (Ingenia CX, Philips Healthcare) using a 32-element phased-array head coil. Three-dimensional pCASL was performed with the following parameters: 3D gradient and spin-echo imaging; 20 sections; 8 control/label pairs, repetition time (TR)/echo time (TE), 3,903/11 ms; SENSE factor, 1.3; labeling duration, 1,800 s; post-labeling delay (PLD), 1.5 s; scan time, 3 min 31 s.

Four-dimensional MRA images were collected based on super-selective pCASL combined with the keyhole and view-sharing techniques (4D-sPACK), which was described in a previous study and used for labeling the external carotid artery (Wang M. et al., 2021). The parameters were the same as those in a previous study, in which scan time was 4 min 52 s (Wang M. et al., 2021). It could be an alternative method for visualization of intracranial collaterals from the external carotid artery after bypass surgery.

Susceptibility-weighted imaging was acquired using the following parameters: 3D fast field echo imaging; TR/TE1/delta TE, 31/7.2/6.2 ms; Echoes, 4; Compressed SENSE factor: 4; voxel size: 0.60 × 0.60 × 2 mm^3^; scan time, 3 min.

### Image Analysis

Arterial spin labeling was quantitatively analyzed as relative cerebral blood flow (rCBF) using Statistical Parametric Mapping software. CBF images were generated from 3D pCASL automatically. The preprocessing was as follows: coregistered CBF images to the corresponding 3D T1-weighted images for each subject, normalized the coregistered T1 images to Montreal Neurological Institute, applied the transformation matrix to CBF images, and then smoothed them with a 6-mm Gaussian kernel. The smoothed CBF image was divided by the averaged CBF of whole cerebral gray matter using Data Processing and Analysis of Brain Imaging. The rCBF values of gray matter in each hemisphere were extracted.

Two neuroradiologists (W.M. and C.F., with 11 and 7 years of experience, respectively) qualitatively analyzed the pre- and postsurgical collateral scores on CBF images by consensus with a 2-week interval. They were blinded to the postsurgical collateral circulation on 4D MRA and mRS. A four-point grading scale was used on two slices of ASL images corresponding to the Alberta Stroke Program Early CT Score locations. The scale was as follows: 0, no or minimal perfusion signal; 1, moderate perfusion signal with arterial transit artifact (ATA); 2, high perfusion signal with ATA; and 3, normal perfusion signal without ATA (Zaharchuk et al., 2011; Lee et al., 2018). Ten regions in each hemisphere were evaluated (a total of 30 scores).

The MRA scores of intracranial arteries were assessed on preoperative maximum intensity projection images by two neuroradiologists (W.M. and C.F., with 11 and 7 years of experience, respectively) according to the criteria in a previous report (Houkin et al., 2005). The ICA score was defined as follows: 0, normal; 1, stenosis of C1; 2, discontinuity of C1 signal; and 3, invisible. The score definition of MCA-M1 was similar to ICA. ACA scores were defined as follows: 0, normal A2 and its distal; 1, A2 and its distal signal decrease or loss; and 2, invisible. The score definition of PCA was similar to ACA. The total MRA score of each hemisphere was 0–10.

Hemorrhage on anastomosis site was assessed on SWI images by one neuroradiologist (W.M. with 11 years of experience). Hemorrhage was considered if hemosiderin deposition was newly found in the anastomosis site after revascularization, which was an irregular patchy low signal on SWI images.

### Follow-Up Evaluation

Follow-up MR exams were collected after more than 3 months. Surgical collaterals were evaluated on super-selective 4D MRA using the Matsushima standard (Zhao et al., 2019): 0–3, null, localized, moderate, and abundant. A score of 0–1 indicated poor surgical collaterals, and a score of 2–3 indicated good surgical collaterals. Evaluations were conducted by a neurosurgeon (W.Y., with 16 years of experience) and a radiologist (C.C., with 4 years of experience), who were blinded to the baseline results. mRS was collected by another neurosurgeon (Y.Y., with 20 years of experience) and another radiologist (W.K., with 10 years of experience), who were blinded to the baseline results and surgical collaterals. The time interval between the MR exam and mRS evaluation was less than 1 week. Disagreements in the qualitative analysis were resolved by consensus.

### Statistical Analysis

Statistical analysis was performed with SPSS 25.0 software (IBM). *P* < 0.05 was considered statistically significant. Interobserver agreement of the collateral score, MRA scores, surgical collaterals, and mRS were assessed using Cohen’s weighted kappa statistic. Kappa values were interpreted as follows: ≤0.20, slight; 0.21–0.40, fair; 0.41–0.60, moderate; 0.61–0.80, substantial; 0.81–1.00, almost perfect. Independent samples *T*-tests and Mann–Whitney U-tests were used to evaluate differences in continuous and categorical variables between the good and poor surgical collateral groups. Paired sample *T*-tests and Wilcoxon signed-rank tests were used to assess the differences between pre- and postsurgical rCBF, collateral score, and mRS. Univariable and multivariable logistic regressions were performed to identify the predictors for surgical collaterals after combined bypass. Two models of multivariable logistic regression were adopted because of the limited sample size. Continuous variables were converted to dichotomous variables according to the largest Youden’s index. The area under the curve was calculated to predict postsurgical collaterals.

## Results

### Subject Characteristics

Pre- and postoperative ASL and 4D MRA were obtained in 63 MMA patients (68 hemispheres). Two hemispheres were excluded because of a cerebral hemorrhage at the anastomosis site. Totally, 61 patients (66 hemispheres) were included. They were divided into good and poor surgical collateral groups according to follow-up results. Five hemispheres in the good surgical collateral group and 10 in the poor group had hemorrhagic onset. The time interval of the preoperative MR exam to bypass surgery and follow-up time were not significantly different between the two groups (Table 1).

**TABLE 1:** Demographic and radiological information of included subjects.

	Surgical collaterals		
	Good (*n* = 29)	Poor (*n* = 37)	*P*
Age (years)	46.59 ± 10.83	47.83 ± 6.58	0.586
Sex (male)	13	23	0.160
**Personal history**			
Hypertension	10	20	0.113
Diabetes	10	11	0.681
Hyperlipidemia	5	12	0.161
Smoking	1	4	0.262
Drinking alcohol	1	3	0.431
**Onset type**			
Hemorrhagic	5	10	0.346
Nonhemorrhagic	24	27	
**Side**			
Right	15	23	0.394
Light	14	14	
MRA score (scores)	4.55 ± 1.97	3.95 ± 1.73	0.216
**ASL**			
rCBF_pre_	0.81 ± 0.13	0.83 ± 0.08	0.639
Collaterals_pre_ (scores)	13.72 ± 7.83	19.16 ± 6.65	0.005
rCBF_post_	0.83 ± 0.11	0.82 ± 0.08	0.682
Collaterals_post_ (scores)	20.79 ± 6.65	22.84 ± 5.06	0.279
Follow-up time (months)	8.07 ± 3.51	7.78 ± 4.06	0.765
Presurgical time interval (days)	4.72 ± 2.74	5.14 ± 2.21	0.502
mRS_pre_	1.66 ± 1.14	1.49 ± 0.90	0.590
mRS_post_	0.52 ± 0.83	0.62 ± 0.76	0.475

*Presurgical time interval: time interval between presurgical MR exam and combined bypass surgery.*

### Presurgical Comparisons

The MRA score was significantly correlated with the presurgical collateral score (*r* = −0.523, *p* < 0.001). The rCBF and mRS in the good surgical collateral group were not significantly different from those in the poor group (rCBF: *P* = 0.639; mRS: *P* = 0.590). However, the poor group had a higher collateral score (19.16 scores ± 6.65) than the good group (13.72 scores ± 7.83) (*P* = 0.005).

### Follow-Up Results

**FIGURE 1:**
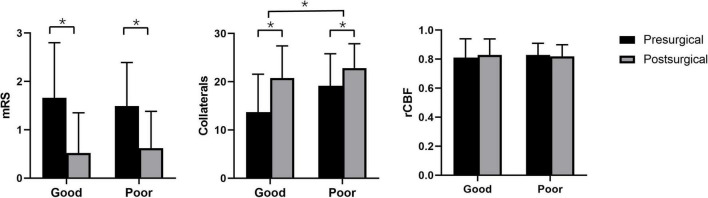
Pre- and postsurgical modified Rankin Scale, collateral perfusion, and relative cerebral blood flow based on arterial spin labeling between good and poor surgical collaterals. *p < 0.05 between two groups.

Postsurgical rCBF was not significantly elevated compared with presurgical rCBF in the good (*P* = 0.119) and poor (*P* = 0.233) groups. Postsurgical collateral scores were significantly elevated in the good (13.72 scores ± 7.83 *vs*. 20.79 scores ± 6.65, *p* < 0.001) and poor (19.16 scores ± 6.65 *vs*. 22.84 scores ± 5.06, *p* < 0.001) groups. Moreover, the elevation of collateral scores in the good group (7.07 scores ± 4.32) was higher than that in the poor group (3.68 scores ± 3.52, *P* = 0.001) (Figure 1). Postsurgical mRS was significantly reduced compared with presurgical (good: 0.52 ± 0.83 *vs*. 1.66 ± 1.14, *P* < 0.001; poor: 1.49 ± 0.90 *vs.* 0.62 ± 0.76, *P* < 0.001) mRS in the good and poor groups.

### Potential Predictors for Surgical Collaterals After Combined Bypass Surgery

**FIGURE 2:**
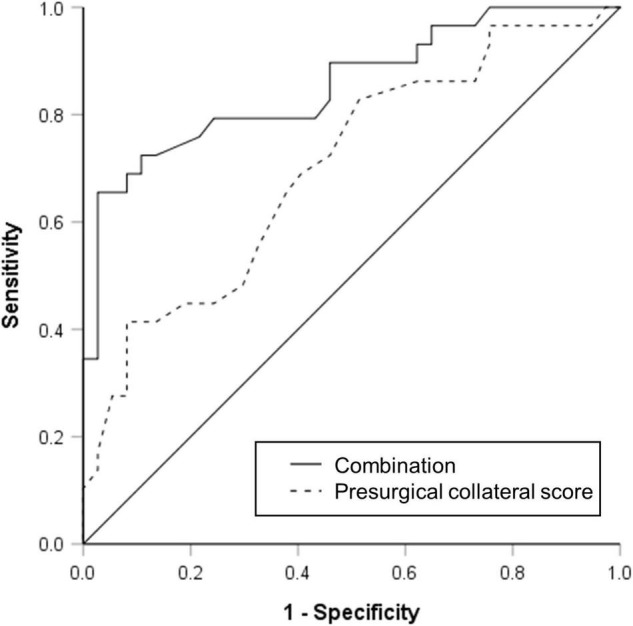
Receiver operating characteristic curve used to evaluate presurgical collateral score (area under curve was 0.702) and combination of presurgical collateral score, sex, age, and hypertension (area under curve was 0.855) for predicting moyamoya angiopathy patients with good surgical collateral circulation after combined bypass surgery.

**FIGURE 3:**
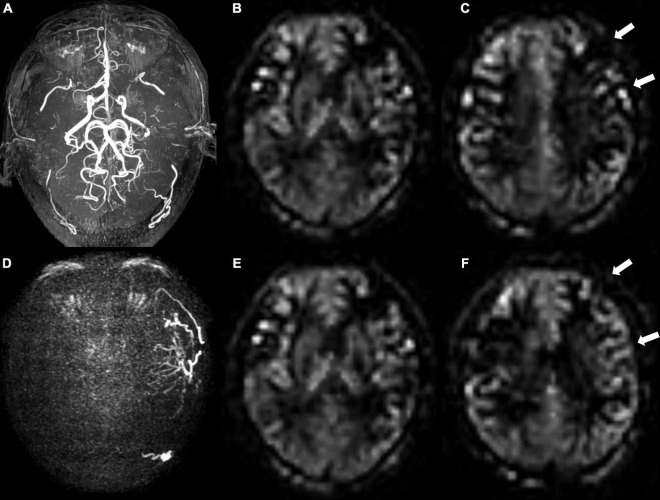
A 48-year-old male patient with moyamoya angiopathy. **(A)** Presurgical MR angiography image. **(B,C)** Two-slice ASL images of ASPECT regions, and preoperative collateral score was 10 in **(B)** and 9 in **(C)**. **(D)** Postoperative super-selective 4D MRA images of external carotid artery. Postsurgical collaterals were defined as poor (1 score according to Matsushima standard). **(E,F)** Same postoperative two-slice ASL images as **(B,C)**. Postoperative collateral scores increased (white arrows) to 10 in **(E)** and 11 in **(F)**.

**FIGURE 4:**
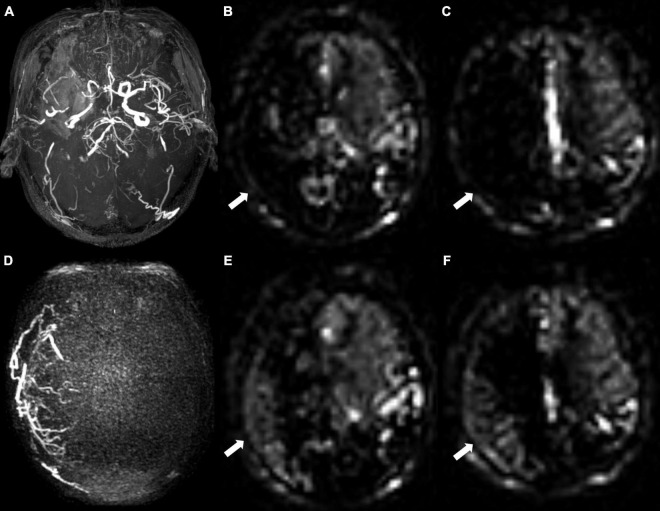
A 68-year-old male patient with unilateral moyamoya angiopathy. **(A)** Presurgical MR angiography image. Right middle cerebral artery was occluded. **(B,C)** Two-slice ASL images of ASPECT regions, and cerebral perfusion was significantly reduced. Preoperative collateral score was almost 0 in **(B)** and 1 in **(C)**. **(D)** Postoperative super-selective 4D MRA images of external carotid artery. Postsurgical collaterals were defined as good (2 scores according to Matsushima standard). **(E,F)** ASL images at same level as **(B,C)**. Postoperative collateral scores increased (white arrows), which were 6 in **(E)** and 6 in **(F)**.

The results of univariable and multivariable logistic regressions are listed in Table 2. Lower presurgical collateral scores (OR: 0.791; 95% CI: 0.695, 0.90; *P* < 0.001), female sex (OR: 0.154; 95% CI: 0.035, 0.676; *P* = 0.013), younger age (OR: 0.181; 95% CI: 0.039, 0.854; *P* = 0.031), and non-hypertension (OR: 0.167; 95% CI: 0.038, 0.736; *P* = 0.018) were associated with good surgical collaterals. The area under the curve of the combined presurgical collateral score, sex, age, and hypertension for predicting surgical collaterals was 0.855 (Figure 2). Figure 3 shows a bilateral MMA patient with poor surgical collaterals, and the collateral score was increased by approximately 2 after 9 months. Figure 4 shows a unilateral MMA patient with good surgical collaterals after 7 months of follow-up. The postsurgical collateral score obviously increased from 1 to 12.

**TABLE 2:** Univariable and multivariable logistic regression results.

	Univariable regression		Multivariable regression			
			Model 1		Model 2	
	OR (95% CI)	P	OR (95% CI)	P	OR (95% CI)	P
Sex	0.495 (0.184, 1.329)	0.163	0.146 (0.031, 0.695)	0.016	0.154 (0.035, 0.676)	0.013
Age	0.317 (0.10, 1.002)	0.05	0.135 (0.026, 0.718)	0.019	0.181 (0.039, 0.854)	0.031
Hypertension	0.447 (0.164, 1.219)	0.116	0.161 (0.033, 0.776)	0.023	0.167 (0.038, 0.736)	0.018
Hyperlipidemia	0.434 (0.133, 1.418)	0.167	0.339 (0.066, 1.729)	0.193		
Diabetes	1.244 (0.439, 3.522)	0.681				
Smoking	0.295 (0.031, 2.791)	0.287				
Drinking	0.405 (0.04, 4.110)	0.444				
Hemorrhage	0.563 (0.168, 1.879)	0.350				
MRA	1.201 (0.914, 1.576)	0.188	0.796 (0.495, 1.281)	0.347		
Side	0.652 (0.243, 1.748)	0.395				
Collaterals_pre_	0.902 (0.838, 0.971)	0.006	0.769 (0.649, 0.912)	0.002	0.791 (0.695, 0.900)	<0.001
rCBF_pre_	0.277 (0.065, 1.188)	0.084	0.650 (0.065, 6.474)	0.713		
Follow-up time	0.739 (0.264, 2.070)	0.565				
mRS_pre_	1.183 (0.726, 1.929)	0.499				

### Reproducibility

Interobserver reproducibility was almost excellent for the assessment of the MRA score (κ = 0.811), surgical collateral circulation (κ = 0.815), and presurgical collateral score (κ = 0.821). The postsurgical collateral score (κ = 0.790) and pre- (κ = 0.764) and postsurgical (κ = 0.786) mRS had substantial interobserver reproducibility.

## Discussion

In this study, we analyzed the characteristics of pre- and postsurgical ASL images in MMA patients who underwent combined bypass surgery to explore the possible role of presurgical ASL in predicting the occurrence of good surgical collateral circulation. We found that the presurgical collateral score was correlated with the MRA score and was lower in patients with good surgical collaterals than in those with poor surgical collaterals. The collateral score increased, and mRS improved after combined bypass surgery. The presurgical collateral score, sex, age, and hypertension may be predictors of surgical collaterals.

The presurgical collateral score based on ASL in this study was evaluated according to the presence of ATA in Alberta Stroke Program Early CT Score areas. ATA is a characteristic artifact in the cerebral cortex and sulcus and is affected by the PLD value (Deibler et al., 2008). ATA on ASL images with a PLD of 1,500 ms has been reported to be correlated with the MRA score in MMA patients. In addition, PLDs of 1,500 ms had a wider range median of the ATA values and a higher correlation coefficient compared with PLDs of 1,000 and 2,000 ms (Ukai et al., 2020). In this study, a PLD of 1,500 ms was adopted in the ASL exam. We also found that the presurgical collateral score based on the ATA was correlated with the MRA score.

Revascularization can improve the clinical outcome in MMA patients with a long median follow-up time (18 and 26.3 months, 12 years) (Duan et al., 2012; Ha et al., 2019; Zhang et al., 2020). In this study, the clinical outcome was improved in all MMA patients, and the postoperative mRS score increased in both good and poor surgical collateral circulation. None of the MMA patients had recurrent cerebrovascular events. This may be due to a shorter median follow-up time of 7 months in our subjects. All MMA patients in our study will be followed in the future continuously.

Collateral circulation is a critical determinant of cerebral perfusion in cerebral ischemia (Liebeskind, 2003). Leptomeningeal collateral circulation plays an important role in the blood supply of ischemic stroke in the anterior cerebral artery and MCA territories in MMA patients (Wang Q. N. et al., 2021). A significantly lower presurgical collateral score on ASL was found in MMA patients with good surgical collateral circulation, which often indicates more severe cerebral hypoperfusion. However, presurgical rCBF in the two groups was not significantly different. This might be because of lower PLD (1,500 ms). Long delay (4,000 ms) and multi-delay ASL can provide more accurate cerebral blood flow in MMA patients compared with positron emission tomography (Fan et al., 2017; Hara et al., 2017). We chose rCBF instead of CBF to reduce the effect of lower PLD in this study. In addition, perfusion differences were calculated using the rCBF of one hemisphere without using an intergroup analysis based on voxels, which may be another reason. The presence of presurgical transdural collaterals trended better surgical collaterals in MMA patients, which also indicated a severe cerebral perfusion defect (Storey et al., 2017). After combined bypass surgery, a higher elevation of the collateral score was found in patients with good surgical collateral circulation.

Younger age was reported to be associated with good surgical collaterals after indirect bypass surgery (Wang Q. N. et al., 2021). It has been demonstrated to be more effective for younger patients than older patients in the long term (Goda et al., 2004). In this study, youth age was also found to be a predictor of good surgical collateral circulation after combined bypass surgery. Moreover, predictors included sex and hypertension. Hypertension was an independent risk factor for unfavorable clinical outcomes and may be a risk factor for decreased bypass patency (superficial temporal artery to MCA) in patients with cerebral atherosclerotic disease (Matano et al., 2016; Ma et al., 2020). This might be the cause of poor surgical collateral circulation. Khan et al. (2012) reported that females might be at a higher risk of postoperative adverse events despite successful bypass surgery in MMA patients. However, in previous studies, sex was not a predictor in MMA patients with good surgical collaterals after indirect bypass surgery (Zhao et al., 2019; Wang Q. N. et al., 2021), which was different from this study. The reason might be the limited sample size.

Several limitations existed in this study. First, different surgeons performing bypass surgery may have an effect on surgical collaterals. The number of surgeons performing bypass surgery in this study was three in the same group. This may need to be confirmed in the future to choose patients from the same surgeon. Second, the current study aimed to explore potential clinical and radiographic predictors based on ASL for good surgical collateral circulation after combined bypass surgery. Some of the other potential biomarkers, such as gene type, were not included. Third, perioperative hyperperfusion reaction was not recorded because this information was not clear in electronic medical records. However, a hemorrhagic event in the anastomosis site was considered according to the deposition of hemosiderin on SWI. Finally, limited sample size was used in this study. We will continuously follow up with more MMA patients after cerebral bypass surgery in the future.

## Conclusion

Patients with good surgical collaterals had lower presurgical collateral scores and higher increased collateral scores on ASL. Lower presurgical collateral scores, youth, female sex, and nonhypertension may indicate good surgical collateral circulation. This may play an important role in improving the management of adult MMA patients and therapeutic decisions.

## Data Availability Statement

The raw data supporting the conclusions of this article will be made available by the authors, without undue reservation.

## Ethics Statement

The studies involving human participants were reviewed and approved by Medical Ethics Committee of the Nanjing Drum Tower Hospital. The patients/participants provided their written informed consent to participate in this study. Written informed consent was obtained from the individual(s) for the publication of any potentially identifiable images or data included in this article.

## Author Contributions

MW, YW, and BZ designed the study. WZ and XZ performed the statistical analyses. MW and YW contributed to data preparation and drafting of the original manuscript. MW was responsible for MR scanning. MW, YW, and YY evaluated radiological and clinical information. YW and YY managed the subject recruitment. BZ and YY modified and confirmed the final article. All authors contributed to the article and approved the submitted version.

## Conflict of Interest

XZ was employed by the company Philips Healthcare, Shanghai. The remaining authors declare that the research was conducted in the absence of any commercial or financial relationships that could be construed as a potential conflict of interest.

## Publisher’s Note

All claims expressed in this article are solely those of the authors and do not necessarily represent those of their affiliated organizations, or those of the publisher, the editors and the reviewers. Any product that may be evaluated in this article, or claim that may be made by its manufacturer, is not guaranteed or endorsed by the publisher.
